# Molecular detection and immunopathological examination of *Deltapapillomavirus* 4 in skin and udder of Egyptian cattle

**DOI:** 10.14202/vetworld.2018.915-920

**Published:** 2018-07-10

**Authors:** Emad Beshir Ata, Mohamed Abd El-Fatah Mahmoud, A. A. Madboli

**Affiliations:** 1 Department of Parasitology and Animal Diseases, Veterinary Research Division, National Research Center, 12622 Dokki, Giza, Egypt; 2 Department of Animal Reproduction and Artificial Insemination, Veterinary Research Division, National Research Center, 12622 Dokki, Giza, Egypt

**Keywords:** bovine papillomaviruses, characterization, Egypt, genotyping, immunohistochemical, pathological, phylogeny

## Abstract

**Aim:**

Bovine papillomaviruses (BPVs) are the main cause of bovine papillomatosis resulting in cutaneous and/or mucosal benign tumors that could be transformed to malignant ones with marked economic importance, especially in the dairy farms. Molecular, pathological, and immunohistochemical (IHC) diagnosis of bovine papillomatosis cases was conducted to identify and characterize the circulating BPV genotype in some Egyptian governorates.

**Materials and Methods:**

Wart-like lesions in skin, udder, and teats were collected from 123 infected cases in Giza, Beni Suef, and El Menoufia Governorates, Egypt, during 2016-2017. Pathological and IHC characterization, molecular identification, genotyping, and phylogenetic analysis based on the conserved late (L1) gene of the all samples were carried out.

**Results:**

89 of the 123 collected samples (72.3%) were positively detected by polymerase chain reaction (PCR). The sequence analysis of the obtained PCR amplicons was identical revealing identification and genotyping of only one type (*Deltapapillomavirus* 4 isolate EGY 2017) with accession number (MG547343) which found to be closely related to the recently detected *Deltapapillomavirus* 4 isolate 04_asi_UK (accession no. MF384288.1) and isolate *Deltapapillomavirus* 4 isolate 25_equ_CH (accession no. MF384286.1) with 99% nucleotide sequence identity. Histopathological examination revealed severe hyperkeratosis in stratum corneum and acanthosis in most of the cases. These tissue changes were confirmed by the presence of golden brown stained proliferating cell nuclear antigen which was localized intranuclear and perinuclear in other cells using IHC Technique.

**Conclusion:**

It is the first time to detect and genotype the BPVs in these areas with no record of previous genotyping in the whole country. The obtained results will highlight the importance of this disease.

## Introduction

The benign skin and mucous membrane warts are mainly caused by the epitheliotropic papillomaviruses (PVs) [1], which are small, non-enveloped, double-stranded DNA viruses with circular genomes [2]. According to the conserved late (L1) gene, the most common 13 BPVs types are found in the four genera *Xipapillomavirus*, *Deltapapillomavirus*, *Epsilonpapillomavirus*, and *Dyoxipapillomavirus* [1,3].

The disease has a worldwide distribution in many areas of the world including America, Europe, and Asia [4,5]. It results in notable economic losses in both of the dairy and beef industries [6]. Teat papillomatosis results in dairy herd problems because of milking process difficulties as a result of ulceration and rupture of the cutaneous lesions which acts as predisposing factors for mastitis and distortion of the milk ducts [7].

Infection to other animals might occur through milk, semen, and urine or vertically transmitted to the offspring [8]. It was reported that coinfection with different BPV types might occur in the same animal [1]. Although PVs are highly host-specific that usually do not cross species barriers, new variants could evolve if mutations accumulate within the virus genome. Highly related PVs were detected in different host species representing the possibility of cross-species infection, which may result in the emergence of new types [9]. The virus can infect different bovine types, but buffalo infection is less common than cattle [10].

The predilection seats of the developed benign cutaneous tumors are the haired skin, teats, penis, and vulva. Hence, the clinical condition of the infected animals varies according to the affected site [11,12].

Identification and molecular characterization of BPV types are important for the disease control. Molecular techniques remain important tools for diagnostic purposes, particularly in determining asymptomatic carriers within the herd [1,13]. Few studies about the situation of this virus in Egypt focus mainly on histopathological examination [14] and different treatment regimens of the resulted surgical wounds [15]. Meanwhile, the virus was successfully isolated on fetal bovine skin tissue culture and embryonated chicken eggs without complete identification, and it was suspected to be BPV of type II [16].

In the present study, molecular, pathological, and immunohistochemical (IHC) diagnosis of bovine papillomatosis cases was conducted to identify and genotype the circulating BPV genotype in some Egyptian governorates.

## Materials and Methods

### Ethical approval

The experiments were carried out in accordance with the guidelines laid down by the National Research Center, Animal Ethics Committee and in accordance with local laws and regulations (NO. 17101).

### Study area and animals samples

**Figure-1:**
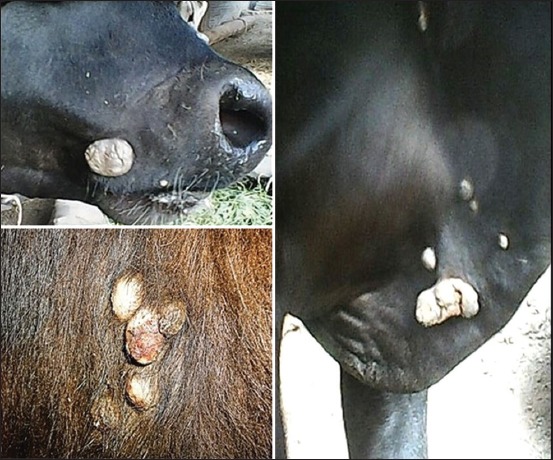
Macroscopic view of wart lesions at different locations of animal bodies.

The samples were collected from eight different farms, six slaughterhouses, and three veterinary clinics at three different Egyptian governorates (Giza, Beni Suef, and El Menoufia) in the period between March 2016 and July 2017. A total of 123 wart lesions were used in this study. The animals under this experiment were of different ages and sex with wart lesions of variable sizes at different locations in the body including head, neck, thighs, udder, and teats (Figure-1). The wart lesion biopsies were collected using local anesthesia 2% lidocaine (lidocaine HCL, Pharco B^®^) under aseptic condition. Each specimen was divided into two portions; the first one was used for molecular identification, while the second portion was used for histopathological and IHC analysis.

### Molecular identification

The viral DNA was extracted from the wart lesion according to the instruction manual of (QIAamp^®^ DNA Mini Kit, Qiagen GmBH). The final eluted DNA was quantified spectrophotometrically and preserved at −20°C for further experiments. The polymerase chain reaction (PCR) conditions were initial denaturation at 95°C for 5 min, followed by 35 cycles consisting of denaturation at 95°C for 30 s, annealing at 55°C for 30 s, and extension at 72°C for 1 min, while the final cycle was elongation at 72°C for 5 min using the MY09 (5-GCMCAGGGWCATAAYAATGG-3) and MY011 (5 CGTCCMARRGGAWACTGATC-3) primers [17]. The PCR products were visualized on ethidium bromide stained 1.5% agarose gel.

### Sequencing and phylogenetic analysis

The obtained PCR amplicons were purified according to the manufacturer’s instructions of the QIAquick PCR Purification Kit, Qiagen, before being sent for sequencing. Bioedit software (Version7.2); (http://bioedit.software.informer.com/7.2/) was used for sequence assembly and editing. The obtained sequence was deposited at the Gene Bank with an accession number (MG547343). The free Basic Local Alignment Search Tool of the National Center for Biotechnology Information https://blast.ncbi.nlm.nih.gov/Blast.cgi was used for the comparison with the similar sequences.

The evolutionary history was inferred using the maximum likelihood method based on the Tamura-Nei model [18]. The analysis involved 29 nucleotide sequences. All positions containing gaps and missing data were eliminated. Evolutionary analyses were conducted using MEGA7 [19]. The confidence level of the tree was assessed by bootstrapping using 1000 replicates.

### Histopathological examination

The collected tissue samples were fixed in 10% neutral buffer formalin (NBF). Tissue specimens were embedded in paraffin, processed, sectioned at the 3-5 µm thickness, and stained with H and E for the detection of the histopathological changes [20].

### IHC examination

Tissue specimens were fixed in 10% NBF overnight, then transferred into ethanol 70% (to keep the antigenicity of the virus particle), then deparaffinized, sectioned at 3 µm thickness, and mounted on positively charged slides. Streptavidin/biotin/peroxidase complex (ABC) detection kit fromScyTek Laboratories, USA, was used. The kit was species specified as anti-mouse and anti-rabbit. Antigen retrieval was done using proteinase K enzyme 0.1%. Tested samples were incubated with rabbit polyclonal anti-BPV1 antibody as anti-proliferating cell nuclear antigen (anti-PCNA) imported from ScyTek Laboratories, USA. Tested sections were faintly counterstained with Mayer’s hematoxylin stain and examined under light microscopy [21].

## Results

### Molecular identification and sequencing analysis

**Figure-2:**
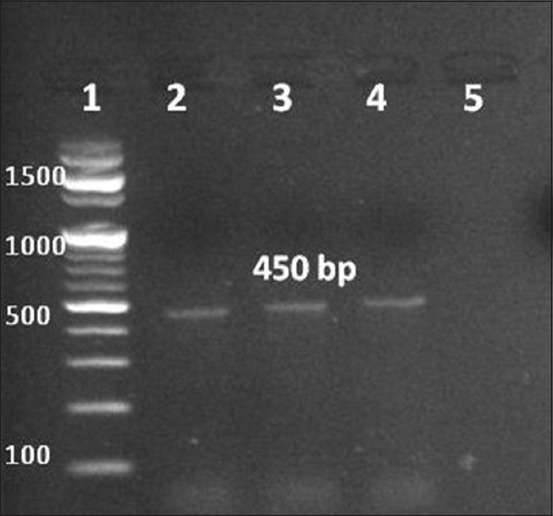
Molecular detection of bovine papillomavirus in bovine wart samples. Partial L1 gene was amplified using My09 and My011 primers set; ethidium bromide stained 1.5% agarose gel electrophoresed in TE buffer was used for visualization. L1: 100 bp Marker (Intron Scientific), L2-L4: specific 450 bp band representing positive samples, and L5: negative sample.

**Figure-3:**
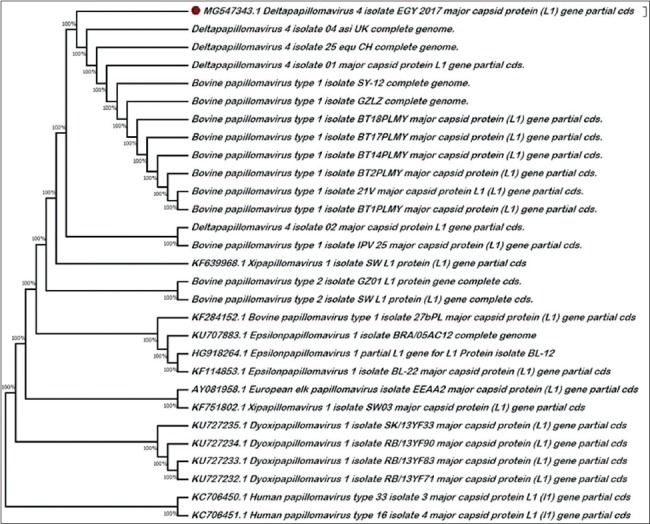
The phylogenetic rooted tree created by MEGA 7 software. The evolutionary history was inferred using the maximum likelihood method based on the Tamura-Nei model. The confidence level of the NJ tree was assessed by bootstrapping using 1000 replicates. The red circle indicates the sequence obtained in this study.

Partial amplification of the L1 gene was successfully detected in 89 of the 123 collected samples (72.3%) with the size of an expected band around 450 bp (Figure-2). The sequence analysis of the obtained PCR amplicons was the identical revealing identification of only one type (*Deltapapillomavirus* 4 isolate EGY 2017). The obtained blast result showed 99% homology with the *Deltapapillomavirus* 4 isolate 04_asi_UK (accession no. MF384288.1), *Deltapapillomavirus* 4 isolate 25_equ_CH (accession no. MF384286.1), and BPV SY-12 strain (accession no. KX271663.1). They were evolved from the same ancestor as shown in the phylogenetic rooted cladogram (Figure-3), the obtained sequences were found to belong to *Deltapapillomaviru*s 4; BPV type-1. To the best of our information, this is the first study to identify and characterize the presence of BPV-1 in Egypt.

### Histopathological findings

**Figure-4:**
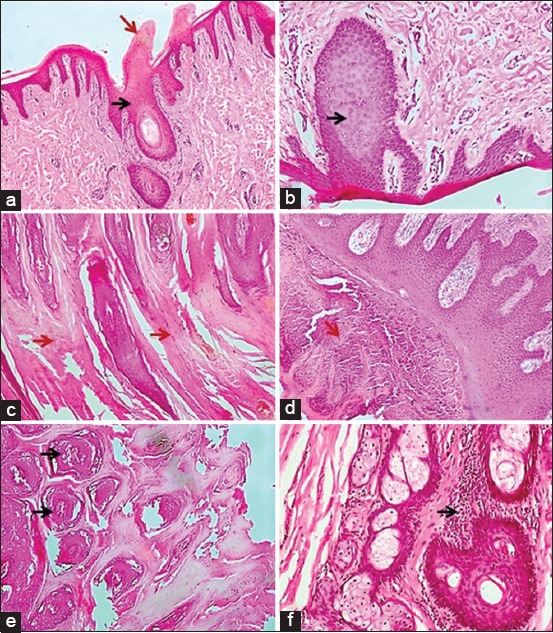
Histopathological examination of the wart lesions. Warts in skin and udder of cows infected with bovine papillomavirus type 1 (BPV-1) showed hyperkeratosis in stratum corneum (red arrow) associated with severe acanthosis in stratum spinosum (black arrow) (a and c: 100×; b: 200×). Severe hyperplasia in the stratum granulosum which invaded by trabeculae came from the corneal layer (red arrow) (d). Multiple hyperplastic masses of the epidermal layer invaded the stratum corneum (black arrows) (e). Moderate focal periglandular aggregation of mononuclear inflammatory cells was found (f: 200×). Tissue specimens stained with hematoxylin and eosin stain.

A total number of 82 tissue samples from the examined wart lesions (123 samples) showed severe hyperkeratosis in stratum corneum which appeared as finger-like projections (Figure-4a) associated with severe acanthosis (increase in the thickness of the stratum spinosum of the epidermis) (Figure-4a-c). In addition to the hyperkeratosis, 48 cases of the 123 examined cases exhibited severe hyperplasia in the stratum granulosum leading to widening of the distance between the corneal layer and the other layers. The areas of overgrowth were invaded by trabeculae from the upper corneal layer (Figure-4d). Other24 cases showed multiple hyperplastic masses in stratum spinosum that invaded the stratum corneum (Figure-4e) and also exhibited moderate focal periglandular aggregation of mononuclear inflammatory cells (Figure-4f).

### IHC findings

**Figure-5:**
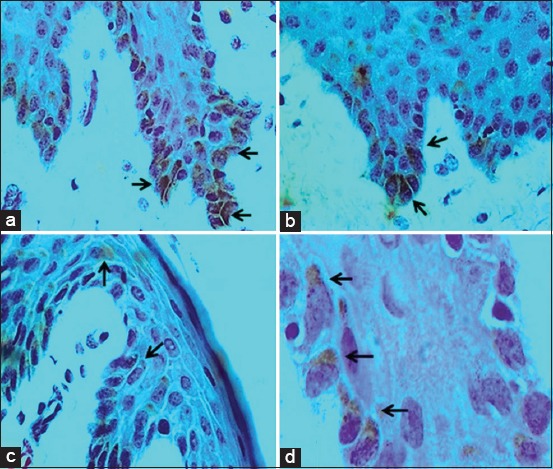
Immunohistochemistry detection of bovine papillomavirus type 1 (BPV-1) proliferating cell nuclear antigen (PCNA). Immunohistochemistry detection of PCNA antigen of BPV-1 by avidin-biotin complex technique was achieved in the examined skin and udder warts. Moderate intensity of golden brown stained BPV antigen was detected intranuclear as in a and b: 400×. In other sites, the BPV antigen found as capping to the nuclear membrane of stratum germinativum and stratum spinosum (c: 400× and d: 1000×). The intensity of viral antigen was high in cells of stratum germinativum more than stratum spinosum. Tissue specimens were counterstained with hematoxylin stain.

Sixty-nine cases of a total number of 123 examined cases showed a strong positive immunoreactive result for the presence of the PCNA antigen of BPV with moderate golden brown intensity using ABC technique. The golden brown stained PCNA antigen was localized intranuclear as shown in Figure-5a and b and perinuclear in other cells as shown in Figure 5c and d in the stratum germinativum and stratum spinosum.

## Discussion

BPVs are classified under the Papillomaviridae family, which infect the epithelium and mucosa of different animals resulting in benign hyperproliferative lesions [3]. Studding of BPV is very important not only due to its economic importance [6,22] but also to the fact that it has represented one of the most extensively studied animal models of viral carcinogenesis, especially, that the resulted benign tumors could be transformed to malignant ones in cases of immune suppression [23,24].

Concerning the obtained results, it was noticed that all of the infected cases were cattle while there were no clinical cases of buffaloes and this agrees with that recorded by Jangir *et al*. [25] and Somvanshi [26]. Although there are many successive trials of identification and characterization of some BPVs in buffalos [27,28], especially that cross-species infection was recorded in cases within mixed herd systems [29].

The lesions were found in different parts of the animal body including teats, haired and hairless areas; this is in accordance with Corteggio *et al*. [11], and Munday [12] who cleared that condition of the infected animal correlates with infected sites.

The percentage of the positive confirmed cases using the MY primer set was 72.3% (89/123), and nearly the same result 77.1% (27/35) was previously recorded by Dagalp *et al*. [1] while 86.42% (121/140) was recorded by Hamad *et al*. [13]. On the other hand, 36% (19/52) and 53% (8/15) of positivity were recorded by Rojas-Anaya *et al*. [10] and Ogawa *et al*. [17], respectively. Although the high sensitivity of the PCR as a diagnostic tool but molecular detection of all samples might not occur, or false negative results could be obtained in case of presence of low copy number of viral DNA or multiple type infections in the same samples [30]. Using of the MY primer set in the detection of PVs in previous studies showed high specificity and sensitivity than the other GP5+/GP6+ [31] or the SPF10 primer sets [32], with superiority in successful detection of multiple PVs types in one sample [31,33]. It was recorded that the sensitivity of the MY set was 90% in some studies which recommended using of such set in the screening of the PVs [31,32].

The sequence analysis of the positive DNA samples revealed the detection of BPV-1 which is the most detected type in different countries [2,34]. It has the ability to infect other species including equine, asinine, and bovine hosts that become a common phenomenon [35]. According to the general criteria and rules used for PV taxonomy, a new genotype is considered if the comparison with the closest strain revealed variance more than 10% of the L1 gene nucleotide sequence [36]. Therefore, the obtained isolate in this study is considered to be *Deltapapillomavirus* 4 as it resembles 99% homologous to the *Deltapapillomavirus* 4 isolate 04_asi_UK strain.

The IHC findings showed that the tissue reaction against BPV-1 infection is proliferative where the main changes arehyperkeratosis in corneal layer and hyperplasia in stratum spinosum and granulosum are confirmed by the detection and localization of PCNA antigen by IHC protocol. Our results come in accordance with Maeda *et al*. [37] who reported that the hallmark lesions of BPV-1 infection are proliferative as epithelial hyperplasia and acanthosis. This proliferation could be explained by the presence of PCNA antigen of BPV which is implicated in the endothelial cell proliferation, angiogenesis, and vascular permeability. Moreover, the underlying skin layer of infected cows contains minute focal aggregations of mononuclear inflammatory cells although the tissue cells are often histologically normal [12].

It is worth noting that genotyping of the PV local strain is highly important, especially that prophylactic vaccines have been used in many parts of the world to prevent and eventually treat infections [38]. The present study is the first one to identify and genotype the presence of BPV-1 in these areas with no record of genotyping in the whole country. The obtained results will highlight the importance and increase the attention and awareness of such infectious disease to implement the best control measures.

## Conclusion

Molecular identification, pathological and IHC characterization, and genotyping of bovine papillomatosis cases were successfully carried out. Amplification of L1 gene deduced 72.3% positivity of the tested samples. Genotyping and phylogenetic analysis revealed identification of *Deltapapillomavirus* 4 for the first time in Egyptian native breed cattle. Using of PCR in the detection of BPV is more sensitive than the histopathological and/or the IHC examination. A whole country well-structured molecular survey using two different sets of primers should be implemented to determine any other types found in Egypt.

## Authors’ Contributions

EBA, AAM, and AAM designed the study and collected the samples. EBA and MAM carried out the molecular diagnosis and the phylogenetic analysis, and MAA conducted the histopathological and IHC examination. All authors analyzed the data, drafted the manuscript, and approved the final manuscript.
